# Alcohol, Oxidative Stress, and Free Radical Damage

**Published:** 2003

**Authors:** Defeng Wu, Arthur I. Cederbaum

**Affiliations:** Defeng Wu, Ph.D., is a research associate professor, and Arthur I. Cederbaum, Ph.D., is a professor, both in the Department of Pharmacology and Biological Chemistry, Mount Sinai School of Medicine, New York, New York

**Keywords:** alcoholic liver disorder, oxidative stress, free radicals, reactive oxygen species, chronic AODE (alcohol and other drug effects), NAD, NADH oxidoreductases, cytochrome P450, peroxidation, metals, proteins, DNA, lipids, glutathione peroxidase, biochemical mechanism, survey of research

## Abstract

Reactive oxygen species (ROS) are small, highly reactive, oxygen-containing molecules that are naturally generated in small amounts during the body’s metabolic reactions and can react with and damage complex cellular molecules such as fats, proteins, or DNA. Alcohol promotes the generation of ROS and/or interferes with the body’s normal defense mechanisms against these compounds through numerous processes, particularly in the liver. For example, alcohol breakdown in the liver results in the formation of molecules whose further metabolism in the cell leads to ROS production. Alcohol also stimulates the activity of enzymes called cytochrome P450s, which contribute to ROS production. Further, alcohol can alter the levels of certain metals in the body, thereby facilitating ROS production. Finally, alcohol reduces the levels of agents that can eliminate ROS (i.e., antioxidants). The resulting state of the cell, known as oxidative stress, can lead to cell injury. ROS production and oxidative stress in liver cells play a central role in the development of alcoholic liver disease.

As described throughout the articles in this issue of *Alcohol Research & Health*, alcohol acts through numerous pathways to affect the liver and other organs and to lead to the development of alcoholic liver disease (ALD) (for summaries of many of these pathways, see Cederbaum 2001; Bondy 1992; Nordmann et al. 1992). No single process or underlying mechanism can account for all the effects of alcohol on an organism or even on one specific organ; instead, many mechanisms act in concert, reflecting the spectrum of the organism’s response to a myriad of direct and indirect actions of alcohol. One factor that has been suggested as playing a central role in many pathways of alcohol-induced damage, and which has been the focus of much research, is the excessive generation of molecules called free radicals, which can result in a state called oxidative stress. (These terms and concepts will be defined and explained in more detail in the following sections.) Particularly important are the actions of a class of oxygen-containing free radicals known as reactive oxygen species (ROS). ROS can damage or cause complete degradation (i.e., peroxidation) of essential complex molecules in the cells, including fat molecules (i.e., lipids), proteins, and DNA. Both acute and chronic alcohol exposure can increase production of ROS and enhance peroxidation of lipids, protein, and DNA, as has been demonstrated in a variety of systems, cells, and species, including humans.

Researchers have learned much about alcohol metabolism and the various enzymes and pathways involved, as well as about the role of lipid peroxidation and oxidative stress in alcohol toxicity. This article summarizes some of these findings. A detailed description of free radicals, ROS, and oxidative stress is followed by a review of the alcohol-related cellular systems involved in ROS production. Next, the article explains why ROS are toxic to cells and what systems have evolved to help cells protect themselves against ROS. Finally, the role of ROS and oxidative stress in alcohol-induced cell injury is discussed, with suggestions about future directions for research in this field. Although this discussion focuses on the role of oxidative stress in alcoholic liver disease, alcohol-induced oxidative stress also occurs in and damages other tissues (e.g., muscle, pancreas, and nerve cells).

## What Are Free Radicals and ROS?

A free radical is an atom, molecule, or compound that is highly unstable because of its atomic or molecular structure (i.e., the distribution of electrons within the molecule). As a result, free radicals are very reactive as they attempt to pair up with other molecules, atoms, or even individual electrons to create a stable compound. To achieve a more stable state, free radicals can “steal” a hydrogen atom from another molecule, bind to another molecule, or interact in various ways with other free radicals (see the textbox).

**Reactions Involving Free Radicals**

Free radicals are highly unstable molecules that attempt to achieve a more stable state by reacting with other atoms or molecules in the cell. The four primary types of chemical reactions that free radicals undergo are:

- *Hydrogen abstraction*, in which a radical interacts with another molecule that has a free hydrogen atom (i.e., a hydrogen donor). As a result, the radical binds to the hydrogen atom and becomes stable, whereas the hydrogen donor is converted to a free radical.
- *Addition*, in which the radical binds to another, originally stable molecule, converting the combined molecule into a radical.
- *Termination*, in which two radicals react with each other to form a stable compound.
- *Disproportionation*, in which two identical radicals react with each other, with one of the radicals donating an electron to the other so that two different molecules are formed, each of which is stable.

One chemical element frequently involved in free radical formation is oxygen. Molecular oxygen (O_2_) is essential for cell function because it plays a pivotal role in a series of biochemical reactions occurring in the respiratory chain, which is responsible for most of the production of adenosine triphosphate (ATP), which provides the energy required for a multitude of cellular reactions and functions. (For more information on the respiratory chain and ATP production, see the article by Cunningham and Van Horn in this issue.)

In the respiratory chain, which takes place in membrane-enclosed cell structures called mitochondria, an electron and a proton (H^+^) are removed from a helper molecule (i.e., cofactor) called reduced nicotinamide adenine dinucleotide (NADH).^1^ The electron is transferred to the first component of the respiratory chain, and the proton is released into the surrounding fluid. Chemically speaking, NADH is oxidized to NAD^+^ in this reaction, whereas the respiratory chain, component that accepts the electron is reduced.^2^ The NAD^+^ subsequently can be used again to accept new hydrogen atoms that are generated during the metabolism of sugars (e.g., glucose) and other nutrients. The reduced respiratory chain component, in turn, passes the electron on to other molecules in the respiratory chain until it is finally transferred to O_2_, which then interacts with protons in cells to generate water. This series of electron transfer reactions generates sufficient energy to produce several molecules of ATP for each electron that passes through the respiratory chain.

Molecular oxygen can accept a total of four electrons, one at a time, and the corresponding number of protons to generate two molecules of water. During this process, different oxygen radicals are successively formed as intermediate products, including superoxide (O_2_^•−^); peroxide (O_2_^=^), which normally exists in cells as hydrogen peroxide (H_2_O_2_); and the hydroxyl radical (^•^OH). Superoxide, peroxide, and the hydroxyl radical are considered the primary ROS and have sparked major research on the role of free radicals in biology and medicine.^3^ However, because they are unstable and rapidly react with additional electrons and protons, most of these ROS are converted to water before they can damage cells. It has been estimated that only about 2 to 3 percent of the O_2_ consumed by the respiratory chain is converted to ROS (Chance et al. 1979). Nevertheless, the toxic effects of oxygen in biological systems—such as the breakdown (i.e., oxidation) of lipids, inactivation of enzymes, introduction of changes (i.e., mutations) in the DNA, and destruction of cell membranes and, ultimately, cells—are attributable to the reduction of O_2_ to ROS (Toykuni 1999; de Groot 1994; Nakazawa et al. 1996).

## What Is Oxidative Stress?

Because ROS form naturally during many metabolic processes, cells have developed several protective mechanisms to prevent ROS formation or to detoxify the ROS. These mechanisms employ molecules called antioxidants, which will be discussed in more detail in the section “Protection Against ROS Toxicity.” Under certain conditions, such as acute or chronic alcohol exposure, ROS production is enhanced and/or the level or activity of antioxidants is reduced. The resulting state— which is characterized by a disturbance in the balance between ROS production on one hand and ROS removal and repair of damaged complex molecules (such as proteins or DNA) on the other—is called oxidative stress (Halliwell 1999). Oxidative stress is associated with numerous deleterious consequences for the cell (e.g., lipid peroxidation or even cell death), and alcohol-induced oxidative stress may play a significant role in the development of ALD.

Many processes and factors are involved in causing alcohol-induced oxidative stress, including:

- Changes in the NAD^+^/NADH ratio in the cell as a result of alcohol metabolism. Alcohol is metabolized in two steps. First, the enzyme alcohol dehydrogenase converts alcohol to acetaldehyde, a toxic and reactive molecule. Next, the enzyme aldehyde dehydrogenase converts the acetaldehyde to acetate. Each of these reactions leads to formation of one molecule of NADH, thereby providing more starting material and thus enhanced activity of the respiratory chain, including heightened O_2_ use and ROS formation.
- Production of acetaldehyde during alcohol metabolism, which through its interactions with proteins and lipids also can lead to radical formation and cell damage. (For information on acetaldehyde and its detrimental effects, see the article in this issue by Tuma and Casey.)
- Damage to the mitochondria resulting in decreased ATP production.
- Effects on cell structure (e.g., the membranes) and function caused by alcohol’s interactions with either membrane components (i.e., phosphate-containing lipids [phospholipids]) or enzymes and other protein components of the cells.
- Alcohol-induced oxygen deficiency (i.e., hypoxia) in tissues, especially in certain areas of the liver lobules (i.e., the pericentral region), where extra oxygen is required to metabolize the alcohol. (For more information on alcohol-induced hypoxia in the liver and its consequences, see the article by Cunningham and Van Horn in this issue.)
- Alcohol’s effects on the immune system, which lead to altered production of certain signaling molecules called cytokines, which in turn lead to the activation of an array of biochemical processes. (For more information on alcohol’s effect on cytokine production and its consequences, see the article in this issue by Neuman.)
- Alcohol-induced increase in the ability of the bacterial molecule endotoxin to enter the bloodstream and liver, where it can activate certain immune cells. (For more information on the role of endotoxin in liver damage, see the article by Wheeler in this issue.)
- Alcohol-induced increases in the activity of the enzyme cytochrome P450 2E1 (CYP2E1), which (as described in the section “Systems Producing ROS”) metabolizes alcohol and other molecules and generates ROS in the process.
- Alcohol-induced increases in the levels of free iron in the cell (i.e., iron that is not bound to various proteins), which can promote ROS generation, as described in the section “Role of Metals.”
- Effects on antioxidant enzymes and chemicals, particularly a molecule called glutathione (GSH), as described in the section “Protection Against ROS Toxicity.”
- Biochemical reactions generating an alcohol-derived radical (i.e., the 1-hydroxyethyl radical).
- Conversion of the enzyme xanthine dehydrogenase into a form called xanthine oxidase, which can generate ROS.

Many of these processes operate concurrently, and it is likely that several, indeed many, systems contribute to the ability of alcohol to induce a state of oxidative stress.

## Systems Producing ROS

As implied in the previous section, numerous cellular systems can produce ROS. The major source of ROS production in the cell is the mitochondrial respiratory chain, which, as described earlier, utilizes approximately 80 to 90 percent of the O_2_ a person consumes. Thus, even though only a small percentage of that oxygen is converted to ROS, the mitochondrial respiratory chain in all cells generates most of the ROS produced in the body.

Another major source of ROS, especially in the liver, is a group of enzymes called the cytochrome P450 mixed-function oxidases. Many different variants of these iron-containing enzymes exist, some of which are responsible for removing or detoxifying a variety of compounds present in our environment and ingested (e.g., foods or drugs), including alcohol. Some cytochrome P450 enzymes also are important for metabolizing substances that naturally occur in the body, such as fatty acids, cholesterol, steroids, or bile acids. The biochemical reactions spurred (i.e., catalyzed) by the cytochrome P450 molecules use molecular oxygen, and during these reactions small amounts of ROS are generated. The extent of ROS generation may vary considerably depending on the compound to be degraded and on the cytochrome P450 molecule involved. One type of cytochrome molecule that is especially active in producing ROS is known as CYP2E1. This enzyme is of particular interest when investigating alcohol-induced oxidative stress because its activity increases after heavy alcohol exposure and because CYP2E1 itself also metabolizes alcohol (Lieber 1997).

ROS also are produced by a variety of oxidative enzymes present in cells, such as the previously mentioned xanthine oxidase. Under normal physiological conditions, xanthine oxidase acts as a dehydrogenase—that is, it removes hydrogen from xanthine or hypoxanthine and attaches it to NAD, thereby generating NADH. However, under certain conditions, such as the disruption of blood flow to a tissue, xanthine dehydrogenase is converted to a ROS-producing oxidase form. Alcohol consumption also may promote the conversion of xanthine dehydrogenase to xanthine oxidase (Sultatos 1988), which can generate ROS, thereby enhancing oxidative stress.

Other sources of ROS in the body are two types of immune cells called macrophages and neutrophils, which help defend the body against invading microorganisms. In this case, however, ROS production is beneficial and even essential to the organism because it plays a central role in destroying foreign pathogens (Rosen et al. 1995). Macrophages and neutrophils contain a group of enzymes called the NADPH oxidase complex, which, when activated, generates superoxide radicals and hydrogen peroxide. Hydrogen peroxide then interacts with chloride ions present in the cells to produce hypochlorite (the active ingredient in bleach), which in turn destroys the pathogen. The NADPH oxidase complex and the resulting ROS production are critical to the body’s defense against all kinds of diseases, as is evident in patients with a condition called chronic granulomatous disease, in which ROS production by the NADPH oxidase complex is drastically reduced. Patients with this condition are highly sensitive to infections and usually die at an early age.

Besides the ROS generation that occurs naturally in the body, humans are constantly exposed to environmental free radicals, including ROS, in the form of radiation, UV light, smog, tobacco smoke, and certain compounds referred to as redox cycling agents, which include some pesticides, but also certain medications used for cancer treatment. The toxicity of these medications against tumor cells (as well as normal body cells) results from the fact that the compounds are modified by cellular enzymes to an unstable intermediate, which then reacts with molecular oxygen to produce the original product plus a superoxide radical. Thus, a vicious cycle of chemical reactions involving these compounds continually produces ROS.

### Role of Metals

Most of the systems for the production of ROS described above produce superoxide radicals or hydrogen peroxide. Earlier studies suggested the possibility that these two radicals could interact with each other to produce the most reactive ROS, the hydroxyl radical (^•^OH). Under normal physiological conditions, direct interaction between these two radicals is not likely to play a significant role in generating hydroxyl radicals. However, in the presence of certain metals, particularly free iron or copper ions, a sequence of two reaction steps can occur that results in hydroxyl radical generation. In the first step, hydrogen peroxide can produce the hydroxyl radical by removing an electron from the participating metal ion.^4^ In the second step, involving the superoxide radical (O_2_^•−^), the original metal ions are regenerated so that they are again available for reaction with the hydrogen peroxide. This combination of two chemical reactions appears to account for most of the hydroxyl radical production in biological systems and explains, at least in part, why metals such as iron and copper produce oxidative stress and ROS-induced injury in cells.

Because of iron’s critical contribution to hydroxyl radical formation, anything that increases the levels of free iron in the cells promotes ROS generation and oxidative stress. Chronic alcohol consumption has been shown to increase iron levels in the body not only when iron-rich alcoholic beverages, such as red wine, are consumed, but also because chronic alcohol consumption enhances iron absorption from food (see Nanji and Hiller-Sturmhöfel 1997). Similarly, adding iron to alcohol-containing diets has been shown to exacerbate liver injury in animal studies (Tsukamoto et al. 1995), whereas administration of agents that capture free iron can prevent or ameliorate alcohol’s toxic effects on the liver (Sadrzadeh et al. 1994).

## Why Are ROS Toxic?

ROS are toxic to cells because they can react with most cellular macromolecules, including proteins, lipids, and DNA.

Proteins perform numerous crucial functions in the cell, primarily in the form of enzymes that mediate most biochemical reactions required for cellular functions. Proteins are made up of approximately 20 different building blocks called amino acids, which differ in their sensitivity to interactions with ROS. For example, the amino acids cysteine, methionine, and histidine are especially sensitive to attack and oxidation by the hydroxyl radical. Accordingly, enzymes in which these amino acids are located at positions that are critical to the enzyme’s activity will become inactivated by the interaction with ROS. Alternatively, the ROS-induced oxidation of proteins can lead to changes in the proteins’ three-dimensional structure as well as to fragmentation, aggregation, or cross-linking of the proteins. Finally, protein oxidation often will make the marked protein more susceptible to degradation by cellular systems responsible for eliminating damaged proteins from the cell.

Lipids that contain phosphate groups (i.e., phospholipids) are essential components of the membranes that surround the cells as well as other cellular structures, such as the nucleus and mitochondria. Consequently, damage to the phospholipids will compromise the viability of the cells. The complete degradation (i.e., peroxidation) of lipids is a hallmark of oxidative damage. The polyunsaturated fatty acids^5^ present in the membranes’ phospholipids are particularly sensitive to attack by hydroxyl radicals and other oxidants. A single hydroxyl radical can result in the peroxidation of many polyunsaturated fatty acid molecules because the reactions involved in this process are part of a cyclic chain reaction. In addition to damaging cells by destroying membranes, lipid peroxidation can result in the formation of reactive products that themselves can react with and damage proteins and DNA. (For more information regarding the actions of such reactive products, see the article by Tuma and Casey in this issue.)

DNA is the cell’s genetic material, and any permanent damage to the DNA can result in changes (i.e., mutations) in the proteins encoded in the DNA, which may lead to malfunctions or complete inactivation of the affected proteins. Thus it is essential for the viability of individual cells or even the entire organism that the DNA remain intact. The building blocks of DNA molecules are called nucleotides; they consist of a sugar component and an organic base. Each DNA molecule consists of two strands of nucleotides held together by weak chemical bonds. Changes in the nucleotides in one strand can result in mismatches with the nucleotides in the other strand, yielding subsequent mutations. ROS are a major source of DNA damage, causing strand breaks, removal of nucleotides, and a variety of modifications of the organic bases of the nucleotides. Although cells have developed repair mechanisms to correct naturally occurring changes in the DNA, additional or excessive changes caused by ROS or other agents can lead to permanent changes or damage to the DNA, with potentially detrimental effects for the cell.

## Protection Against ROS Toxicity

Because ROS production is a naturally occurring process, a variety of enzymatic and nonenzymatic mechanisms have evolved to protect cells against ROS (Yu 1994). At least some of these mechanisms are impaired after long-term alcohol consumption and may therefore contribute to damage to the liver and other organs.

### Protective Enzymes

Enzymes involved in the elimination of ROS include superoxide dismutases (SODs), catalase, and glutathione peroxidase. SODs catalyze the rapid removal of superoxide radicals. In mammals there are several types of SODs, which differ with respect to their location in the cells and the metal ions they require for their function. For example, a copper–zinc SOD is present in the fluid filling the cell (i.e., the cytosol) and in the space between the two membranes surrounding the mitochondria. Furthermore, a manganese-containing SOD is present in the mitochondrial interior (i.e., matrix). Both of these enzymes are critical for prevention of ROS-induced toxicity (Fridovich 1997).^6^ The effects of chronic alcohol exposure on the cellular content or activity of SODs are controversial, with reports of increases, no changes, or decreases, depending on the model, diet, amount, and time of alcohol feeding. Studies employing a commonly used model in which alcohol is administered directly into the stomach of laboratory animals (i.e., the intragastric infusion model, used most commonly with rats and mice) found decreases in SOD activity in the liver (Polavarapu et al. 1998) (see the article by Nanji and French in this issue).

Catalase and the glutathione peroxidase system both help to remove hydrogen peroxide. Catalase is an iron-containing enzyme found primarily in the small membrane-enclosed cell components called peroxisomes; it serves to detoxify hydrogen peroxide and various other molecules. One way that catalase eliminates hydrogen peroxide is by catalyzing a reaction between two hydrogen peroxide molecules, resulting in the formation of water and O_2_. In addition, catalase can promote the interaction of hydrogen peroxide with compounds that can serve as hydrogen donors so that the hydrogen peroxide can be converted to one molecule of water, and the reduced donor becomes oxidized (a process sometimes called the peroxidatic activity of catalase). Compounds that can provide these hydrogen atoms include beverage alcohol (i.e., ethanol) and methanol.

The glutathione peroxidase system consists of several components, including the enzymes glutathione peroxidase and glutathione reductase and the cofactors glutathione (GSH) and reduced nicotinamide adenosine dinucleotide phosphate (NADPH).^7^ Together, these molecules effectively remove hydrogen peroxide. GSH, which consists of three amino acids, is an essential component of this system and serves as a cofactor for an enzyme called glutathione transferase, which helps remove certain drugs and chemicals as well as other reactive molecules from the cells. Moreover, GSH can interact directly with certain ROS (e.g., the hydroxyl radical) to detoxify them, as well as performing other critical activities in the cell.

### Nonenzymatic Mechanisms

Because of all its functions, GSH is probably the most important antioxidant present in cells. Therefore, enzymes that help generate GSH are critical to the body’s ability to protect itself against oxidative stress. Alcohol has been shown to deplete GSH levels, particularly in the mitochondria, which normally are characterized by high levels of GSH needed to eliminate the ROS generated during activity of the respiratory chain.

Mitochondria cannot synthesize GSH but import it from the cytosol using a carrier protein embedded in the membrane surrounding the mitochondria. Alcohol appears to interfere with the function of this carrier protein, thereby leading to the depletion of mitochondrial GSH (Fernandez-Checa et al. 1997).

NADPH is involved in a much more diverse range of reactions in the cell than GSH. Nevertheless, because of its role in the glutathione peroxidase system, NADPH or the enzymes that generate this compound are sometimes considered antioxidants.

In addition to GSH and NADPH, numerous other nonenzymatic anti-oxidants are present in the cells, most prominently vitamin E (α-tocopherol) and vitamin C (ascorbate). Vitamin E is a major antioxidant found in the lipid phase of membranes and, like other chemically related molecules, acts as a powerful terminator of lipid peroxidation. During the reaction between vitamin E and a lipid radical, the vitamin E radical is formed, from which vitamin E can be regenerated in a reaction involving GSH and ascorbate. Alcohol also appears to interfere with the body’s normal vitamin E content because patients with ALD commonly exhibit reduced vitamin E levels (see Nanji and Hiller-Sturmhöfel 1997).

## Alcohol, Oxidative Stress, and Cell Injury

Excess levels of ROS and the resulting oxidative stress have been implicated in a variety of human diseases (see the sidebar). What is the evidence that alcohol-induced oxidative stress plays a role in cell injury, particularly damage to the liver cells? Many studies have demonstrated that alcohol increases lipid peroxidation as well as the modification of proteins; however, it is not always clear if these changes are the causes rather than consequences of alcohol-induced tissue injury. Nevertheless, numerous investigations have found that administering antioxidants, agents that reduce the levels of free iron, or agents that replenish GSH levels can prevent or ameliorate the toxic actions of alcohol. For example, in the intragastric infusion model, the antioxidant vitamin E; the chemical ebselen, which mimics the actions of glutathione peroxidase; the copper–zinc or manganese SODs; or a GSH precursor—all prevented ALD (Iimuro et al. 2000; Nanji et al. 1996; Kono et al. 2001; Wheeler et al. 2001a,b).

**Diseases Involving Excessive ROS Levels**

In addition to contributing to the development of ALD, ROS have been implicated in many other major diseases that plague humans. A partial listing of these conditions (Knight 1998; Kehrer 1993) includes:

- The toxic effects of O_2_ itself, such as the oxidation of lipids and proteins, generation of mutations in the DNA, and destruction of cell membranes.
- Cardiovascular diseases.
- Atherosclerosis.
- Various types of cancer.
- Diabetes.
- Neurodegenerative diseases, including Parkinson’s disease and Alzheimer’s disease.
- Toxicity of heavy metals (e.g., iron).
- Radiation injury.
- Vitamin deficiency.
- Toxicity of certain medications.
- Inflammation, such as the destruction of joints, the synovial fluid that lubricates joints and one of its components (i.e., hyaluronic acid), as well as activation of inflammation-promoting signaling molecules called cytokines.
- Toxic effects of tobacco smoke.
- Emphysema.
- Cataracts.

Finally, increasing evidence suggests that aging may be a consequence of the normal, long-term exposure to ROS and the accumulation of oxidized, damaged molecules within the cell—a process that could be likened to a lifetime of “rusting away.”

Accordingly, the health benefits of administering antioxidants such as vitamins E and C or other compounds are the subject of much current research, and clinical trials employing antioxidants in the treatment of various conditions are under way. For example, some therapeutic interventions with antioxidants have shown success or promise in the treatment of Parkinson’s disease and in reducing the toxicity of the cancer medication adriamycin.

Not all instances of ROS production are detrimental to the organism, however. One beneficial effect, as the main article describes, is the production of ROS by certain immune cells in order to destroy invading foreign organisms (Rosen et al. 1995). Furthermore, recent evidence suggests that ROS, especially hydrogen peroxide, may be important in signal transduction mechanisms in cells and thus may be an integral component of cellular physiology and metabolism (Lander 1997).

—Defeng Wu and Arthur I. Cederbaum

ReferencesKehrerJPFree radicals as mediators of tissue injury and diseaseCritical Reviews in Toxicology2321481993847115910.3109/10408449309104073KnightJAFree radicals: Their history and current status in aging and diseaseAnnals of Clinical and Laboratory Science2833134619989846200LanderHMAn essential role for free radicals and derived species in signal transductionFASEB Journal1111812419979039953RosenGMPouSRamosCLFree radicals and phagocytic cellsFASEB Journal92002091995754015610.1096/fasebj.9.2.7540156

The most convincing data indicating that oxidative stress contributes to ALD come from studies using the intragastric infusion model. In these studies, ALD was associated with enhanced lipid peroxidation, protein modification, formation of the 1-hydroxyethyl radical and lipid radicals, and decreases in the hepatic antioxidant defense, particularly GSH levels (Knecht et al. 1995; Tsukamoto and Lu 2001; Iimuro et al. 2000; Nanji et al. 1994; Morimoto et al. 1994). Moreover, changes in the animals’ diets that helped promote or reduce oxidative stress led to corresponding changes in the extent of liver injury. For example, when polyunsaturated fats (which are required for lipid peroxidation to occur) were replaced with saturated fats or other types of fats (i.e., medium-chain triglycerides), lipid peroxidation as well as ALD were reduced or prevented completely, indicating that both alcohol and polyunsaturated fats must be present for ALD to occur. The extent of the ALD was further exacerbated when iron—which, as mentioned earlier, is required for the generation of the hydroxyl radical and therefore promotes oxidative stress—was added to these diets (Tsukamoto et al. 1995). Conversely, the addition of antioxidants such as vitamin E, SOD, or GSH precursors prevented the development of ALD, as mentioned above.

In addition to these studies conducted with intact animals (i.e., in vivo), studies with liver cells (i.e., hepatocytes) grown in culture also showed that alcohol can produce oxidative stress and hepatocyte toxicity. Studies with hepatocytes isolated from control rats or from rats that continuously had been fed alcohol indicated that alcohol metabolism via the enzyme alcohol dehydrogenase results in increased ROS production, hepatocyte injury, and a type of cell death known as apoptosis. Moreover, all of these reactions could be blocked by the administration of antioxidants (Adachi and Ishii 2002; Bailey and Cunningham 2002). Finally, studies using an established hepatocyte cell line that contains the alcohol-metabolizing and ROS-producing enzyme CYP2E1 demonstrated that adding alcohol, polyunsaturated fatty acids, or iron, as well as reducing GSH, resulted in cell toxicity, increased oxidative stress, and mitochondrial damage (Wu and Cederbaum 1999). Furthermore, all of these reactions could be prevented by administering antioxidants. Taken together, these findings indicate that alcohol-induced oxidative stress is a pivotal factor in the development of ALD.

## Future Directions for Research

Although researchers already have gained substantial insight into the mechanisms and consequences of alcohol-induced oxidative stress, additional studies are required to further clarify how alcohol produces oxidative stress in various tissues. For example, more detailed information is needed on the mechanisms involved in some of the major proposed pathways (e.g., how alcohol-derived NADH leads to ROS production either directly or during the passage of NADH-derived electrons through the mitochondrial respiratory chain). Other mechanisms remain highly controversial, such as the role of CYP2E1 or of various cytokines in alcohol-induced oxidative stress. Additional analyses need to determine the role of alcohol metabolism and its byproducts (e.g., acetaldehyde) in the production of ROS. Finally, it still is unclear how alcohol-induced oxidative stress is produced in tissues where only limited alcohol metabolism occurs.

Many of these issues can be studied using animal models; however, extrapolation of findings from animals to humans will be a difficult task because ROS production and antioxidant status in humans are affected by numerous nutritional, environmental, and drug influences that are difficult to reproduce in animals. To date, scattered data suggest that the blood of human alcoholics can contain lipids modified by radicals and other reactive molecules as well as immune molecules targeted at such modified lipids and proteins. These data indicate that ROS and other reactive molecules are indeed formed in human alcoholics. (For more information on the presence of such compounds in humans, see the article by Tuma and Casey in this issue.)

Other questions that should be addressed in future research include the following:

- Do reactive nitrogen species (e.g., nitric oxide) play a role in alcohol-induced oxidative stress in addition to ROS?
- What is the impact of possible interactions between alcohol and environmental influences such as smoking, use of other drugs or medications, and viral infections (e.g., hepatitis C) on ROS production, oxidative stress, and tissue injury? These interactions must be better defined because most alcoholics are exposed to one or more of these influences in addition to alcohol.
- How is oxidative stress affected by interactions between alcohol and nutritional factors, such as the levels and specific types of fats ingested? And how much iron is “safe” in a heavy drinker?
- What are the effects of antioxidants (e.g., vitamin E, vitamin C, or carotenoids) in heavy drinkers? This question is important because some antioxidants can be toxic under certain conditions.

The ability of alcohol to promote oxidative stress and the role of free radicals in alcohol-induced tissue injury clearly are important areas of research in the alcohol field, particularly because such information may be of major therapeutic significance in attempts to prevent or ameliorate alcohol’s toxic effects. As basic information continues to emerge regarding the role of oxidative stress in disease development and the mechanisms underlying ROS-related cellular toxicity, these findings will lead to more rational antioxidant therapeutic approaches. Moreover, these findings could result in the development of more effective and selective new medications capable of blocking the actions of ROS and, consequently, the toxic effects of alcohol.

## Footnotes

1 NADH is generated in the fluid filling the cell (i.e., the cytosol) and then moves to the mitochondria.

2 Oxidation reactions are those that add oxygen to a molecule or remove hydrogen or an electron from a molecule. The reverse reactions (i.e., removal of oxygen or addition of hydrogen or electrons) are called reductions.

3 Superoxide can react with itself to produce H_2_O_2_. Thus, systems producing superoxide also will result in formation of H_2_O_2_. Technically, H_2_O_2_ is not a free radical, but it is commonly included among the ROS.

4 This reaction can generate other products as well, but the hydroxyl radical appears to be the primary oxidant generated (McCord 1998).

5 Unsaturated fatty acids are those that contain a double bond between two of the carbon atoms making up the backbone of the fatty acid molecule. These double bonds can easily be opened in chemical reactions and interact with other substances. Fatty acids containing only one such double bond are called monounsaturated; fatty acids with two or more double bonds are called polyunsaturated.

6 Another type of SOD (EC–SOD) is found outside the cells.

7 Glutathione peroxidase contains an amino acid that is modified by addition of a molecule of the metal selenium; therefore, low amounts of selenium are critical for the body’s antioxidant defense.
